# Construction and Application of Standardized Postoperative Pain-Management Procedure for Patients With Perianal Abscess: A Retrospective Study

**DOI:** 10.3389/fsurg.2022.809622

**Published:** 2022-07-18

**Authors:** Xiu-Mei Wang, Wei-Lian Jiang, Li-Fang Ma, Yue Guo, Li-Ping Cui, Yan-Bin Niu

**Affiliations:** ^1^ Department of Central Surgery Department, Shanxi Bethune Hospital, Shanxi Academy of Medical Sciences, Tongji Shanxi Hospital, Third Hospital of Shanxi Medical University, Shanxi, China; ^2^ Department of the Operating Room, The Second Affiliated Hospital of Guilin Medical University, Guilin, China; ^3^ Department of Hospital Infection Office, Shanxi Bethune Hospital, Shanxi Academy of Medical Sciences, Tongji Shanxi Hospital, Third Hospital of Shanxi Medical University, Shanxi, China; ^4^ Department of Nursing Department, Shanxi Bethune Hospital, Shanxi Academy of Medical Sciences, Tongji Shanxi Hospital, Third Hospital of Shanxi Medical University, Shanxi, China

**Keywords:** perianal abscess, pain, standardized procedures, nursing, postoperative pain

## Abstract

**Objective:**

The present study explored the construction and application of a standardized postoperative pain-management procedure for patients with perianal abscess.

**Methods:**

Two study groups (the observation group and the intervention group) were established retrospectively. The observation group comprised 46 patients with perianal abscess who enrolled in this study between June 2019 and June 2020. The intervention group comprised 48 patients who enrolled in the study between July 2020 and July 2021. All patients were enrolled using the convenience sampling method. A pain-management team was established, and standardized procedure management was implemented in the intervention group, while routine pain management was implemented in the observation group. Indices related to the patients' postoperative pain-control satisfaction and rehabilitation were compared between the two groups.

**Results:**

The patients' pain-control satisfaction, wound edema score, edema disappearance time, urinary retention, and defecation difficulty following intervention were better in the intervention group than in the observation group, and the differences were statistically significant (*P* < 0.05 for all).

**Conclusion:**

The implementation of the standardized postoperative pain-management procedure in patients with perianal abscess can effectively improve the patient's level of pain and satisfaction and promote rehabilitation.

## Introduction

The incidence of postoperative pain in patients with perianal abscess is almost 100%, with most pain being at a moderate to severe level (1, 2). Postoperative pain may give rise to a series of problems, such as the fear of eating and defecating. This may lead to defecation difficulty, urinary retention, electrolyte disorder, and even anal fistula and sepsis; these issues can seriously affect both patient recovery and quality of life (3, 4).

In 2018, the Chinese Medical Association's pain branch proposed that “standardized management is the sole way for pain management” (5). The standard operation procedure (SOP) refers to the description of the standard steps and requirements of a certain operation in a unified format as well as the formation of guidelines and procedures to help navigate and standardize daily care. At the core of SOP is the refinement and quantification of the program's key control points (6).

As an effective intervention procedure, SOP has been used for the prevention and control of infectious respiratory disease (7), microsurgical nursing cooperation (8), and intravenous therapy (9), with good results. To effectively improve the postoperative pain of patients with perianal abscess, Guilin hospital introduced the SOP in 2020. Using the postoperative pain characteristics of patients with perianal abscess, a standardized pain-management procedure was constructed and applied in clinical practice, with good results. The details are reported below.

## Subjects and Methods

### Subjects

This case–control retrospective study was conducted in a tertiary hospital in Guilin, Guangxi, China. Two study groups (the observation group and the intervention group) were established. Patients in the observation group received routine pain management, and patients in the intervention group were treated under the standardized pain-management procedure on the basis of routine pain management. All patients were enrolled using the convenience sampling method.

The inclusion criteria were (1) patients who met the diagnostic criteria of perianal abscess and had surgical indications (10), (2) patients aged 18–70 years, and (3) patients who volunteered to participate in the study.

The exclusion criteria were (1) patients with anemia, heart failure, and severe arrhythmia, or patients who suffered a cerebrovascular accident, (2) patients with serious liver and kidney insufficiency, (3) patients with mental illness, and (4) patients who were pregnant or lactating.

### Methods

#### Intervention Methods in the Intervention Group

In addition to the implementation of routine nursing methods, the following procedures were used in the intervention group.

##### Establishing a Postoperative Pain-Management Team for Patients with Perianal Abscess

The management team was organized jointly by the nursing department and the gastrointestinal surgical medical staff. The director of the nursing department and the head nurse for gastrointestinal surgery were in charge. The team had 10 members, including one director of the nursing department, one director and one deputy director of gastrointestinal surgery, one head nurse, and six doctors and nurses. Of these, five had senior professional titles, two had intermediate professional titles, and three had primary professional titles.

The director of the nursing department was responsible for supervising the implementation of the project, and the head nurse was in charge of formulating the specific implementation plan and summarizing the data. The charge nurses were responsible for the quality control of the implementation process and the specific implementation of the scheme. Additionally, the charge nurses provided feedback. A weekly feedback meeting was held to solve operational problems promptly and improve the quality of the process.

##### Personnel Training and Assessment

To ensure the scheme's smooth implementation, the team leader organized weekly 30-minute training sessions for the research team members in the form of theoretical teaching, on-site demonstrations, scenario simulations, and group discussions. The training was conducted a total of three times.

After the training was completed, theory and operation examinations with a full score of 100 points were organized. A score of ≥85 points indicated that the staff member was qualified to perform the procedure. The pass rate of the entire staff's examinations was 100%.

Before the implementation of the scheme, the team leader formulated a scheme implementation management system; they also established a WeChat group to facilitate the timely responses to questions and coordinate the resolution of implementation process problems. Joint team discussions were conducted to formulate the standard procedure for postoperative pain management for patients with perianal abscess as an operational flow chart, which was placed beside the patient’s bed. The responsible nurse implemented the scheme and authorized the relevant pain management according to the procedure.

Regular monthly meetings were organized in which the members of the research team, the postoperative pain-management team for patients with perianal abscess, and the scheme implementation personnel discussed and summarized the problems and difficulties occurring in the implementation process to continually optimize the process and enhance its quality control.

##### Construction of a Standardized Postoperative Pain-Management Procedure for Patients with Perianal Abscess

Previous studies have shown that the level of early postoperative pain catastrophization is higher in patients with hemorrhoids (11, 12). In patients with hemorrhoids, acute postoperative pain can be summarized into six themes: pain catastrophization belief, persistent negative thoughts about pain, perceived inability to cope with pain, expectation of social support, in addition to painkillers, and anticipation of Traditional Chinese Medicine (TCM) nursing technology (11, 12). The data were retrieved from the British Medical Journal Best Practice database, the Australian Evidence-based Health Care Center database, Cochrane Library, Web of Science, China HowNet, Wanfang, and other databases. A standard pain-management procedure for patients after perianal abscess surgery was established in accordance with the requirements stated in the “Clinical Practice Guidelines for Common Pain Management of Perianal Abscess” (13). The procedure was created on the basis of SOP and research group discussions (see Supplementary Table S1). In general, more psychological counseling; more guidance on diet, activity, and medication; more treatment based on TCM, and less painful operations were implemented in the intervention group.

#### Intervention Methods in the Observation Group

Patients in the observation group received routine nursing. For postoperative pain management in patients with perianal abscess, the responsible nurses provided mainly oral bedside health education (supplemented by the health education manual, WeChat telephone follow-up, and other methods) to (1) educate patients on the significance of pain management, correctly guide their diet, urination, defecation, and activities, and inform them to take diclofenac sodium sustained-release tablets orally (75–150 mg, once a day, prescribed by an anesthetist) or (2) use empathy for pain relief.

According to the frequency specified by the hospital, the responsible nurses visited the patients' bedsides at 8:00 and 16:00 every day to implement the visual analog scoring method and facial expression scale for patient pain evaluation. These scores, along with the analgesic effect, were recorded in detail. Before dressing changes, the responsible nurses additionally fumigated and washed the wound using TCM to achieve an anti-inflammatory, analgesic, and wound-cleaning effect and arranged routine dressing changes by doctors.

#### Evaluation Indices

(1) Pain control and satisfaction: The Houston Pain Outcome Instrument was used, which was sinicized by Chinese scholar Qu Shen in 2006 and tested for reliability and validity (14). A study (15) revealed that a questionnaire can be used as an evaluation tool for postoperative pain management and achieving pain-control satisfaction. The scale consists of 3 subscales, including the impact of pain on the body or daily life (5 items), satisfaction with pain control or relief methods (6 items), and satisfaction with pain-control education (6 items) (total of 17 items). Each item was scored using a digital scoring method from 0 to 10 points with a total score of 170 points; the lower the score of the impact of pain on daily life, the better the pain control, while the higher the satisfaction score, the better the satisfaction.
(2) Patient rehabilitation indicators: The patient rehabilitation indicators were wound edema incidence, edema disappearance time, urinary retention, defecation difficulty, and other complications. For the wound edema score, a four-grade scoring method was used (16): 0 points = no edema, 1 point = mild edema (<1/4 perianal area, with mild edema and skin lines), 2 points = moderate edema (1/4–1/2 perianal edema, with slow capillary filling but no obvious dermatoglyph), and 3 points = severe edema (edema area >1/2 perianal edema, with dermatoglyphics, capillary filling disappearance, and shiny skin).

#### Data-Collection Method and Quality Control

The responsible nurse established the patient's file upon their enrolment in the present study and completed the general patient information according to the patient's medical record. The responsible nurse recorded data during the intervention, and the remaining information was collected by the research team personnel responsible for data collection according to the actual patient evaluation results.

The wound edema data included information on the wound edema immediately after operation and at 3, 5, and 7 days after operation; the edema disappearance time was recorded when the patient returned to the hospital for a follow-up one month after the operation. If the patient is discharged 5 to 7 days after surgery, the nurse in charge will guide the patient to evaluate wound edema. To ensure information validity, the research subjects were selected in strict accordance with the standards, and the researchers used unified guidelines during data collection and skillfully implemented the intervention process into the patient's routine.

#### Statistical Analysis

The statistical data analysis was conducted using SPSS 19.0 software. The count data were expressed as the number of cases and percentage and were compared using an chi-squared test. The measurement data were expressed as mean ± standard deviation (x ± SD) and compared using a t-test. The inspection level was set at *α* = 0.05.

## Results

### Baseline Characteristics

From June 2019 to June 2020, 46 patients with perianal abscess in our hospital were included in the observation group. These patients received routine pain management. Between July 2020 and July 2021, 48 patients with perianal abscess were included in the intervention group.

The observation group (*n* = 46) comprised 30 males and 16 females aged 26–58 years (37.41 ± 5.24 years). The education level was primary school or junior middle school in 16 patients, senior high school or technical secondary school in 19 patients, and junior college or above in 11 patients. The patients' body mass index (BMI) was 18.3–27.6 (24.86 ± 2.15), and the course of the disease was 3–9 days (4.86 ± 1.34 days). The space abscess type was an ischiorectal abscess in 10 cases, a low perianal abscess in 16 cases, a posterior anorectal abscess in 11 cases, and a pelvirectal abscess in 9 cases.

The intervention group (*n* = 48) comprised 33 males and 15 females aged 28–29 years (37.69 ± 5.63 years). The education level was primary school or junior middle school in 18 patients, senior high school or technical secondary school in 18 patients, and junior college or above in 12 patients. The patients' BMI was 18.9–27.6 (24.95 ± 2.34), and the course of the disease was 3–10 days (5.12 ± 1.43 days). The space abscess type was an ischiorectal abscess in 10 cases, a low perianal abscess in 15 cases, a posterior anorectal abscess in 12 cases, and a pelvirectal abscess in 11 cases.

The differences in gender, age, educational level, BMI, course of disease, and space abscess type between the two groups were not statistically significant (*P* > 0.05). Therefore, the two groups were comparable (Table 1).

**Table 1:** Baseline characteristics of the two groups.

Parameters	Observation group (*n* = 46)	Intervention groups (*n* = 48)	t /Z/*χ*^2^	*P*-value
Gender (*n*, %)				
Male	30 (65.22)	33 (68.75)		
Female	16 (34.78)	15 (31.25)	1.634	0.187
Age (mean ± SD)	37.41 ± 5.24	37.69 ± 5.63	0.897	0.329
Degree of education (*n*, %)				
Primary and Junior Secondary	16 (34.78)	18 (37.50)		
Senior high school	19 (41.30)	18 (37.50)		
Junior college or above	11 (23.91)	12 (25.00)	1.032	0.193
BMI index (mean ± SD)	24.86 ± 2.15	24.95 ± 2.34	0.736	0.412
Hospital stay days (day ± SD)	4.86 ± 1.34	5.12 ± 1.43	1.738	0.176
Type of perianal abscess (*n*, %)				
Ischiorectal space abscess	10 (21.74)	10 (20.83)		
Low perianal abscess	16 (34.78)	15 (31.25)		
Posterior anorectal space abscess	11 (23.91)	12 (25.00)		
Supralevator abscess	9 (19.57)	11 (22.92)	0.869	0.335

*BMI, Body Mass Index; SD, standard deviation.*

### Comparison of Postoperative Pain Impacts and Satisfaction Scores Between the Two Groups

The impact score of postoperative pain on the body or daily life after the intervention was lower in the intervention group than in the observation group, and the patient satisfaction with pain control and methods of pain control or relief was higher in the intervention group than in the observation group. The differences were statistically significant (*P* < 0.005 for all, Table 2).

**Table 2:** Comparison of postoperative pain impact and satisfaction scores between the two groups ($\bar{x}\pm S$, scores).

Groups	Observation group (*n* = 46)	Intervention group (*n* = 48)	*t*	*P-*value
The impact of pain on the body or daily life	35.63 ± 4.12	26.85 ± 4.03	7.964	0.003
Pain control education satisfaction	36.89 ± 4.24	45.41 ± 4.27	8.127	0.001
Satisfaction with pain control or relief methods	41.13 ± 3.52	52.03 ± 4.41	8.368	0.000

### Comparison of Postoperative Wound Edema Scores Between the Two Groups

There was no significant difference in the wound edema score immediately after operation between the two groups (*P* > 0.05). However, at 3, 5, and 7 days after operation, the wound edema score was better in the intervention group than in the observation group; the differences were statistically significant (*P* < 0.05, Table 3).

**Table 3:** Comparison of postoperative wound edema scores between the two groups ($\bar{x}\pm S$, scores).

Groups	Observation group (*n* = 46)	Intervention group (*n* = 48)	*t*	*P-*value
Immediately after operation	1.48 ± 0.40	1.50 ± 0.42	0.941	0.639
On postoperative day 3	1.27 ± 0.31	1.10 ± 0.30	4.396	0.006
On postoperative day 5	1.09 ± 0.30	0.88 ± 0.24	6.387	0.003
On postoperative day 7	0.98 ± 0.35	0.71 ± 0.21	7.241	0.000

### Comparison of Edema Disappearance Time, Incidence of Urinary Retention, and Defecation Difficulty After Intervention Between the Two Groups

The edema disappearance time was 11.03 ± 1.32 days in the intervention group and 16.36 ± 1.46 days in the observation group; the difference between the two groups was statistically significant (t = 7.698, *P* < 0.005). The incidences of urinary retention and defecation difficulty were 4.17% (2/48) and 8.33% (4/48), respectively, in the intervention group and 10.87% (5/46) and 19.57% (9/46), respectively, in the observation group; the differences between the two groups were statistically significant (*χ*^2^ = 6.874, 7.137, *P* < 0.005) (Table 4).

**Table 4:** Comparison of edema resolution time and complications between the two groups after intervention.

Groups	Observation group (*n* = 46)	Intervention group (*n* = 48)	t/*χ*^2^	*P*-value
Edema resolution time (days ± SD)	16.36 ± 1.46	11.03 ± 1.32	7.698	0.001
Uroschesis (*n*,%)	10.87% (5/46)	4.17% (2/48)	6.874	0.004
Difficult defecation (*n*,%)	19.57% (9/46)	8.33% (4/48)	7.137	0.003

*Note: SD, standard deviation.*

## Discussion

### Implementation of the Standardized Postoperative Pain-Management Procedure for Patients with Perianal Abscess is Conducive to Pain Outcome and Patient Satisfaction Improvement

The peripheral anal canal is innervated mainly by the pudendal nerve from the spinal nerve and is sensitive to pain. Surgical trauma will increase the sensitivity of the central and peripheral nervous system, increase the release of pain-causing substances (such as histamine, 5-hydroxytryptamine, and plasma carnosine), and stimulate the pain nerve. Pain is one of the main problems faced by postoperative patients.

A previous study (17) revealed that the incidence of moderate and severe postoperative pain in patients with perianal abscess was as high as 65%; moreover, some problems, such as poor pain evaluation and insufficient pain management, occurred in the study. Li Guilan et al. (18) applied predictive nursing to patients with postoperative pain of perianal abscess; the approach can significantly reduce pain and improve patient satisfaction. There was a lack of pain nursing measures targeting perianal pain characteristics (defecation, movement, and dressing change) (19). Yuqin Wang et al. (20) stated that the advantage of a standardized procedure lies in the formulated measures having specific and clear quantitative standards, which make for a targeted and planned nursing operation.

In the present study, the standard pain-management procedure was formulated based on the clinical practice guidelines of perianal abscess pain management, and self-management and TCM techniques were used during key time periods (defecation and dressing change). The procedure can help understand the key time points of pain health education and encourage and support patients in pain self-management. Furthermore, continuous monitoring, timely analysis, and quality improvement were undertaken to ensure the quality of pain management, thus effectively improving patients' pain outcomes and improving their satisfaction.

The results of the present study revealed that the pain control and satisfaction scores after intervention were significantly better in the intervention group than in the observation group; the differences were statistically significant (*P* < 0.05).

### Implementation of the Standardized Postoperative Pain-Management Procedure in Patients with Perianal Abscess can Promote Effective Rehabilitation

The anal canal is surrounded by an abundance of blood vessels and nerves. Patients with perianal abscess are prone to developing postoperative anal edema, urinary retention, and defecation difficulty. The most effective way to address pain management is to establish a pain-management program for a disease. Studies have found that the establishment and implementation of perioperative pain-management programs for patients with intracranial tumor (21) and postoperative pain-management programs for patients with lumbar spine (22) have achieved good pain-management outcomes. However, there are few reports about the postoperative pain management of perianal abscess.

A standardized procedure for pain management based on standard procedures and the requirements of pain-management guidelines during the accelerated rehabilitation of perianal abscess was formulated and implemented in the present study. The procedure provides individualized management from a patient's perspective, and its construction gradually forms a nursing procedure in the process of continuous practice and exploration. Not only is the work content detailed, the key links prominent, and the steps clear, but the concepts of advanced pain management and TCM nursing are also integrated into the procedure.

Pain management is integrated into the patient's education on the first day of admission. Communication and counseling are provided one day before the operation, self-management and TCM techniques are used on the first day after the operation, and supervision and encouragement are given on days 2–5 after the operation.

The results of the present study revealed that the wound edema score, edema disappearance time, urinary retention, and defecation difficulties after the implementation of the standardized pain-management procedure were better in the intervention group than in the observation group (*P* < 0.05).

## Conclusion

In the present study, a pain-management team was established to formulate and implement a standardized procedure for postoperative pain management for patients with perianal abscess; this effectively improved the patients’ level of pain and satisfaction and promoted patient rehabilitation. Ultimately, good results were achieved.

However, the results may be biased due to the relatively small study sample and the rapid turnover of patients with perianal abscess. Sample size expansion and continuous monitoring are required to further verify and improve the scheme. At the same time, the implementation process of this study focuses on pain management during defecation. The impact of perianal abscess type on postoperative pain was not analyzed. In future work, we will continue to explore appropriate pain evaluation methods and multidimensional evaluation tools for patients with perianal abscess after surgery, further improve the pain-management program, and greatly improve the implications of pain care.

## Data Availability

The raw data supporting the conclusions of this article will be made available by the authors, without undue reservation.

## References

[1] Sahnan K Adegbola SO Tozer PJ Watfah J Phillips RK. Perianal abscess. Br Med J. (2017) 356:j475. 10.1136/bmj.j475. 28223268

[2] Zhang JJ Gao HX Zhang TT Bao WQ Mou JY Meng K Pre-mixed nitrous oxide/oxygen mixture treatment of pain induced by postoperative dressing change for perianal abscess: study protocol for a randomized, controlled trial. J Adv Nurs. (2020) 76(12):3623–30. 10.1111/jan.14515 32951241

[3] Gkegkes ID Stamatiadis AP. Anal pain of an unusual cause: role of endoanal ultrasound. J Med Ultrasound. (2019) 27(2):107. 10.4103/JMU.JMU_90_18 31316223 PMC6607880

[4] La Torre M Lisi G D'Agostino E Boccuzzi M Campanelli M Varriale M Lift and VAAFT for high trans-sphincteric anal fistula: a single center retrospective analysis. Int J Colorectal Dis. (2020) 35(6):1149–53. 10.1007/s00384-020-03584-0 32300885

[5] Liu HX. [Standardized management is the only way for the development of pain discipline – an interview with Professor Zhang Daying, chairman designate of pain credit association of Chinese Medical Association.]. China Med News. (2018) 33(13):17. (In Chinese). 10.3969/j.issn.1000-8039.2018.13.013

[6] Ward LC. Bioelectrical impedance analysis for body composition assessment: reflections on accuracy, clinical utility, and standardisation. Eur J Clin Nutr. (2019) 73(2):194–9. 10.1038/s41430-018-0335-3. 30297760

[7] Abbott TEF Fowler AJ Pelosi P Gama de Abreu M Møller AM Canet J A systematic review and consensus definitions for standardised end-points in perioperative medicine: pulmonary complications. Br J Anaesth. (2018) 120(5):1066–79. 10.1016/j.bja.2018.02.007. 29661384

[8] Society of Prosthodontics, Chinese Stomatological Association. [Standard operating procedure for microscopic tooth preparation]. Chin J Stomatol. (2021) 56(4):318–23. 10.3760/cma.j.cn112144-20210225-00089. 33832031

[9] Wang H Zhang JL Cai WX. [Application of standard operating procedures in pre laboratory quality management of venous blood samples.]. Chin J Pract Nurs. (2019) 35(20):1567–70. (In Chinese). 10.3760/cma.j.issn.1672_7088.2019.20.010

[10] Clinical Guidelines Committee, Colorectal Surgeons Branch of Chinese Medical Doctor Association. [Consensus of Chinese experts on the diagnosis and treatment of anal fistula (2020)]. Zhonghua Wei Chang Wai Ke Za Zhi. (2020) 23(12):1123–30. 10.3760/cma.j.cn.441530-20200925-00537. 33353263

[11] Jiang WW Gong X Lu M Tang HJ Zhou Z Li YT [Status and influencing factors of early pain disaster in patients after mixed hemorrhoids surgery]. Jie Fang Jun Hu Li Za Zhi. (2021) 38(9):79–81. (In Chinese). 10.3969/j.issn.1008-9993.2021.09.020

[12] Gong X Tang HJ Jiang WL Zhou Z Li YT [Qualitative study of postoperative acute pain beliefs in patients with mixed hemorrhoids]. Hu Li Xue Bao. (2021) 28(18):56–60. (In Chinese). 10.16460/j.issn1008-9969.2021.18.056

[13] Henderson KG Wallis JA Snowdon DA. Active physiotherapy interventions following total knee arthroplasty in the hospital and inpatient rehabilitation settings: a systematic review and meta-analysis. Physiotherapy. (2018) 104(1):25–35. 10.1016/j.physio.2017.01.002. 28802773

[14] Shen Q Li Z. [Reliability and validity of Houston pain questionnaire.]. Chin J Nurs. (2006) 41(11):1049–51. (In Chinese). http://med.wanfangdata.com.cn/Paper/Detail?id=PeriodicalPaper_zhhlzz200611036&dbid=WF_QK

[15] Weiss SL Peters MJ Alhazzani W Agus MSD Flori HR Inwald DP Surviving sepsis campaign international guidelines for the management of septic shock and sepsis-associated organ dysfunction in children. Intensive Care Med. (2020) 46(Suppl):10–67. 10.1007/s00134-019-05878-6. 32030529 PMC7095013

[16] Amato A Bottini C De Nardi P Giamundo P Lauretta A Realis Luc A Evaluation and management of perianal abscess and anal fistula:SICCR position statement. Tech Coloproctol. (2020) 24(2):127–43. 10.1007/s10151-019-02144-1 31974827

[17] Dolinger MT Person H Smith R Jarchin L Pittman N Dubinsky MC Pediatric crohn disease and multisystem inflammatory syndrome in children (MIS-C) and COVID-19 treated with infliximab. J Pediatr Gastroenterol Nutr. (2020) 71(2):153–5. 10.1097/MPG.0000000000002809 32452979 PMC7268863

[18] Li GL. [Effect of predictive nursing on postoperative pain of perianal abscess]. He Bei Yi Yao. (2018) 40(15):2384–7. (In Chinese). 10.3969/j.issn.1002-7386.2018.15.040

[19] Chen M Li Q. [Application of TCM nursing clinical pathway in perianal abscess operation]. Zhong Xi Yi Jie He Hu Li. (2018) 4(3):64–6. (In Chinese). 10.11997/nitcwm.201803018

[20] Wang YQ Liu YP Wang YL Zheng W Chen X. Construction and practice of pain management standard process for patients with hepatic artery chemoembolization. Chin J Nurs. (2020) 55(4):558–63. 10.3761/j.issn.0254-1769.2020.04.015

[21] Xu ZF Hong SH Wang SJ Zhao X Liu YY Ding SS Neuroendocrine-immune regulating mechanisms for the anti-inflammatory and analgesic actions of acupuncture. World J Tradit Chin Med. (2020) 6:384–92. 10.4103/wjtcm.wjtcm_41_20

[22] Xu HP Zhao H Li DZ Guan LN Mei ZZ Zhang CL [Formulation and application of intelligent pain management program for patients after lumbar spine surgery]. Zhong Hua Hu Li Za Zhi. (2020) 55(10):1465–70. (In Chinese). 10.3761/j.issn.0254-1769.2020.10.004

