# Supplementary material for: Construction and Application of Standardized Postoperative Pain-Management Procedure for Patients With Perianal Abscess: A Retrospective Study
Source: Front Surg. 2022 Jul 18;9:809622. doi: 10.3389/fsurg.2022.809622 (PMC9339645; doi:10.3389/fsurg.2022.809622)
Supplement: Supplementary file 1 [file Table_1_v1.doc]

**Supplementally table 1:** standardized postoperative pain management procedure for patients with perianal abscess.

|  | Nursing strategy | Contents |
| --- | --- | --- |
| On the 1^st^ day of admission | Knowledge education | (1) On the first day, the hospital distributed "Health Education Manual of Mixed hemorrhoids in The Department of Anorectal Medicine"; (2) Patients log on to the wechat official account of the department to understand disease-related knowledge, watch propaganda and education videos, and answer questions on the spot; (3) Explain relevant knowledge: Introduce the etiology and dietary precautions of mixed hemorrhoids to patients from the aspects of occupation and living habits, and inform patients that if they are excessively worried and nervous, the operation of qi and blood can be blocked, and the dysfunction of viscera can aggravate their condition. |
| On preoperative day 1 | Communication and counseling | Nurses employ communication skills such as inquiry, listening and empathy to communicate frequently with patients, encourage patients to fully express their inner thoughts and feelings during pain, and the methods and effects of pain self-management, to understand their physical and psychological state, and dredge them in time; to properly point out the misunderstanding of patients' pain cognition, for example, some patients think that oral painkillers will become addicted and they would become dependent on drugs, and dare not take oral painkillers; refusing medical treatment for fear of pain, resulting in decades of delay in medical treatment, to record the patient's problems, and intervene the individual problems during follow-up; to introduce the operation and anesthesia methods, operation principle, operation effect, precautions during operation, the purpose and precautions of dietary guidance, preoperative examination, preoperative preparation, and the importance of preoperative sleep to patients, according to the anesthesia method, to guide the time of fasting of food and water, and to guide the training of patients to urinate and defecate in bed; to inform patients to carry out painless ward management mode, to standardize the whole process painless operation procedures, and achieve painless treatment in the whole process (during outpatient examination, admission operation, postoperative dressing change, discharge review) to the greatest extent. The details are as follows: (1) During outpatient and admission examination, in principle, the examination methods causing pain shall not be used as much as possible; (2) the patients are fumigated and washed with traditional Chinese medicine according to their conditions before operation; (3) analgesic drugs such as intravenous drip or intramuscular injection of lornoxicam and parecoxib sodium, or oral administration of diclofenac sodium and paracetamol oxycodone, are used according to the doctor’s advice after operation, those with severe pain shall be given intramuscular injection of pethidine hydrochloride; (4) after operation, traditional Chinese medicine techniques such as TCM fumigation and washing, TCM ironing, wrist ankle acupuncture, beans embedding at ear acupoints, TCM dressing, TCM plugging and so on can be given to enhance the confidence to overcome the disease. |
| On postoperative day 1 | Self management and TCM technique | It is found in clinical practice that, generally, the pain is time-dependent, and the patient basically exhibit pain during defecation and dressing change: (1) Pain nursing during defecation is mainly treated by self-management. A, The diet should be bland, digestible, high in vitamins and crude fibers, and patients should drink plenty of water, patients avoid overeating after operation, and avoid spicy and fried food, liquid and semi liquid foods can be eaten 24 hours after operation, and gas producing foods (such as milk and bean products) are prohibited to prevent abdominal distension, soft food or common food can be taken on the second day after operation, and an appropriate amount of dietary fiber can be increased to avoid the formation of constipation and aggravate the pain after defecation; B. To guide patients to develop the habit of regular defecation, and do not control diet because of fear of defecation, so as to avoid water electrolyte disorder due to insufficient diet. (2) Pain nursing during dressing change is mainly treated with TCM technique, twice a day, 16 minutes each time, prescription drugs based on TCM, such as heat clearing and detoxifying herbs, blood cooling and hemostasis herbs, detumescence and pain relieving herbs are used. The patient is in a sitting position, to expose the hips. 1/2 dose of TCM granules is taken and fully dissolved with 60 ml of warm boiled water, and then poured into the laser hip bath machine. The water temperature is adjusted to 39-41°C. About 2,000 ml of water is added to the laser sitting bath machine and heated and the water temperature is kept constant. The patients are instructed to sit their hips in the bidet of the laser sitting bath machine. First, the affected area is fumigated with hot steam for 5 minutes, then washed with bubbles and warm TCM liquid and massaged with the bubble for 10 minutes. After the end of cleaning, it is automatically dried with hot air for 1 minute, a total of 16 minutes. |
| On postoperative day 2-5 | Supervision and encouragement | According to the patients' knowledge of disease and pain, to supplement the blind spots of missing knowledge, give supervision and encouragement, and strengthen the stability of patients' behavior change. It is mainly carried out in the form of daily ward rounds, nurse-patient exchange meetings, regular reexamination, etc., for example, let the patients explain to the patients who are still at a lower stage their own way of relaxation during pain attack, the method and effect of pain self-management, failure and reflection, and the support given by the family at the nurse-patient communication meeting or regular review, to establish an effective information and family support system, to give supervision and encouragement to better strengthen the patient's behavior and prevent the patient from falling back to the low level due to symptom relief and relaxation of self-management of pain. |
